# Supplementary figures and images for: Lipid Nanoparticles Outperform Electroporation in Delivering Therapeutic HPV DNA Vaccines
Source: Vaccines (Basel). 2024 Jun 17;12(6):666. doi: 10.3390/vaccines12060666 (PMC11209142; doi:10.3390/vaccines12060666)

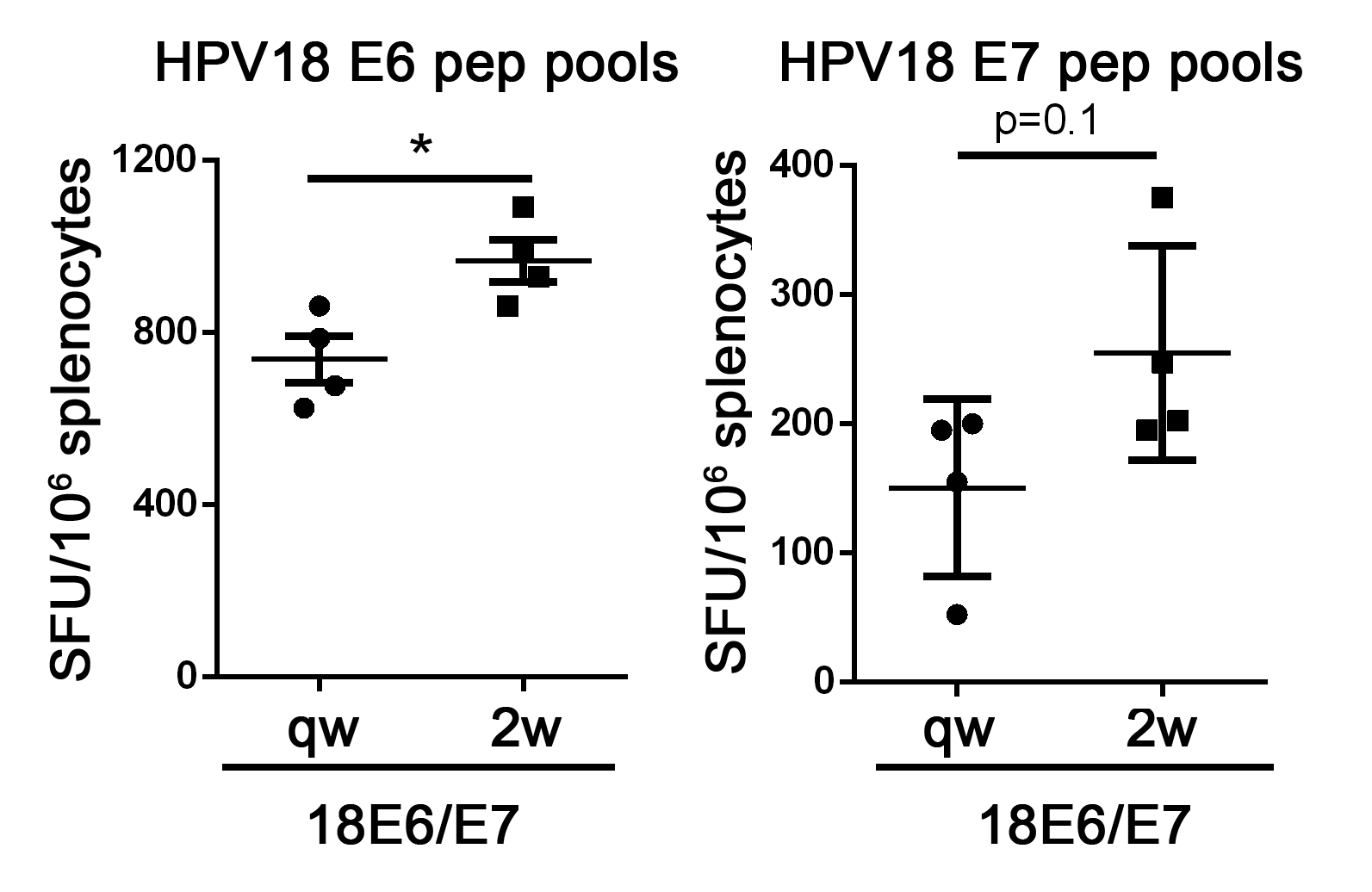

Supplement: Supplementary file 1 [file vaccines-12-00666-s001.zip › Fig S1.tif]

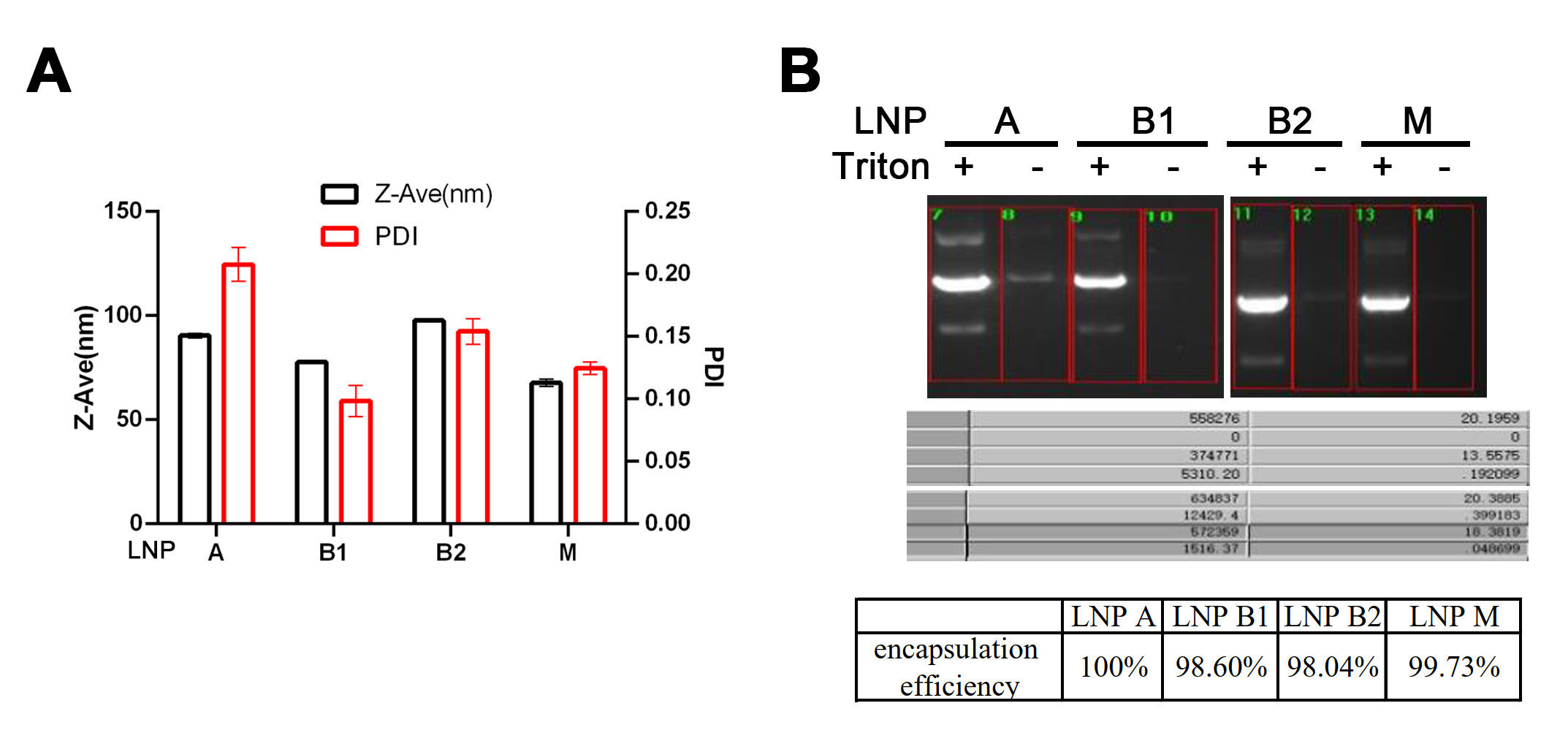

Supplement: Supplementary file 1 [file vaccines-12-00666-s001.zip › Fig S2.tif]

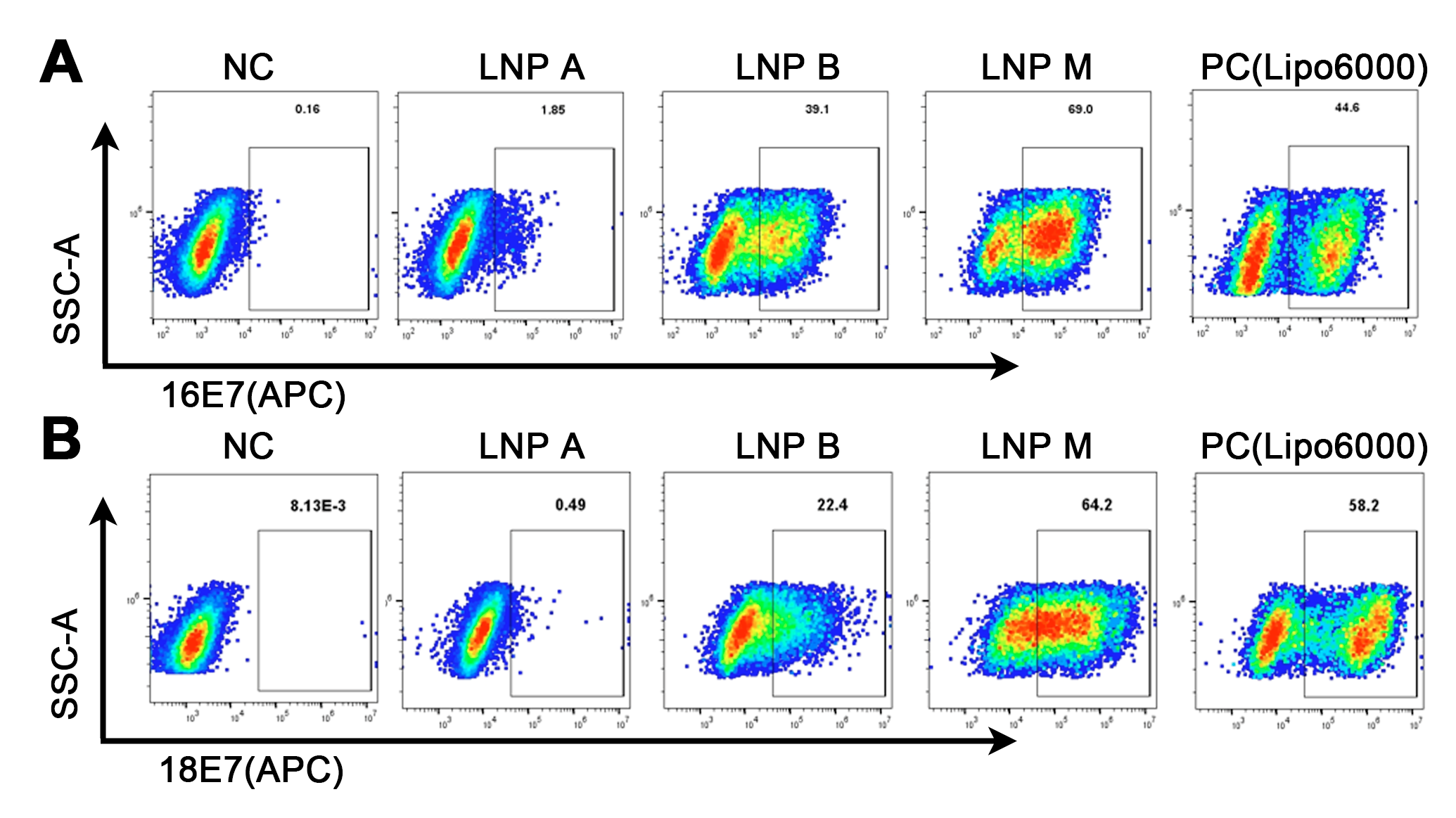

Supplement: Supplementary file 1 [file vaccines-12-00666-s001.zip › Fig S3.tif]
